# A Comparative Study of Nalbuphine, Clonidine, and Dexmedetomidine as Adjuvants to Ropivacaine in Epidural Anesthesia for Lower Limb Surgery

**DOI:** 10.7759/cureus.75507

**Published:** 2024-12-10

**Authors:** Usha Shukla, Manoj Kumar, Deepanshu Panchal, Deepika Doneria

**Affiliations:** 1 Anesthesiology and Critical Care, Uttar Pradesh University of Medical Sciences, Etawah, IND

**Keywords:** 0.75% ropivacaine, clonidine, dexmedetomidine, epidural anesthesia, lower limb orthopedic surgery, nalbuphine

## Abstract

Background: In epidural anaesthesia, the addition of an adjuvant to local anaesthetics enhances the efficacy, thereby providing increased duration and intensity of blockade in lower limb surgeries. The aim was to compare the efficacy, onset, and duration of sensory and motor blockade; haemodynamic changes; and sedative and analgesic effects of nalbuphine, clonidine, and dexmedetomidine as an adjuvant to ropivacaine in epidural anaesthesia.

Methodology: A prospective, randomised, double-blind study among 90 patients after taking consent was divided into three groups (30 patients each; Group D received 15 ml of 0.75% ropivacaine + injection (inj.) dexmedetomidine 1.5 μg/kg + normal saline to make it a total volume of 18 ml; Group N received 15 ml of 0.75% ropivacaine + inj. nalbuphine 0.2 mg/kg + normal saline to make it a total volume of 18 ml; Group C received 15 ml of 0.75% ropivacaine + inj. clonidine 1.5 μg/kg + normal saline to make it a volume of 18 ml, given epidurally). Data were analysed using IBM SPSS Statistics software version 23.0 (IBM Corp., Armonk, NY), and appropriate statistical tests were applied.

Results: The onset of sensory and motor block in Group D was 8.0±1.1 minutes and 10.5±1.7 minutes, respectively; in Group C, 10.3±1.4 minutes and 14.7±1.1 minutes, respectively; and in Group N, 11.3±1.5 minutes and 14.8±1.4 minutes, respectively, found to be very highly statistically significant (p<0.001). The total duration of sensory, motor block and analgesia was longest in Group D (495.5±16.1 minutes, 405.7±16 minutes, and 525.5±16.1 minutes, respectively), followed by Group N (356.8±17.7 minutes, 257±13.4 minutes, and 386.8±17.6 minutes, respectively), and shortest in Group C (309.9±13.4 minutes, 255.7±11 minutes, and 340.0±13.4 minutes, respectively), found to be very highly statistically significant. (p < 0.001). Out of 30 patients, 12 patients in Group D, six in Group C, and eight patients in Group N had sedation (Ramsay's Sedation Score (RSS) > 3). The clonidine group showed significant bradycardia, hypotension, nausea, and vomiting as compared to the others.

Conclusion: We concluded that the use of dexmedetomidine as an adjuvant to the local anaesthetic agent during epidural block hastens the onset of sensory and motor blockade, provides a longer duration of sensory and motor block, provides longer duration of analgesia, and decreases the total analgesic requirement without causing clinically significant and unmanageable side effects as compared to nalbuphine followed by clonidine.

## Introduction

In the past 25 years, there has been a significant evolution and improvement in both surgical and anaesthetic techniques. Various methods and medication regimens have been tried occasionally to reduce anxiety and relax patients during regional anaesthesia, with varying degrees of success [1]. The orthopaedic procedure can cause severe perioperative pain, and improper analgesia is a cause of prolonged discharge and unexpected hospital admission. Management of acute postoperative pain following major orthopaedic surgery is an essential part of perioperative anaesthetic practice to improve patient comfort and to facilitate early rehabilitation [2]. In lower limb procedures, epidural anaesthesia is a frequently utilised anaesthesia approach for both surgical anaesthesia and postoperative analgesia.

Local anaesthetics, “adjuvants” or “additives", contribute in their own special manner to potentiate the analgesic effect of the local anaesthetics [3]. By increasing the duration of sensory-motor block and limiting the total dose requirement of local anaesthetics, co-administration of adjuvants has the potential to raise the standard of nerve blocks and decrease local anaesthetic toxicity.

The United States FDA has approved ropivacaine as an amide local anaesthetic. Ropivacaine is less lipophilic and has a lower propensity to pierce big myelinated motor fibres. Ropivacaine is also manufactured as a pure S (-) enantiomer. The S (-) enantiomer is considerably less cardiotoxic and neurotoxic; an adjuvant can reduce the overall amount of local anaesthetic required and increase its efficacy, but ropivacaine may still be needed in somewhat larger doses. This results in longer-lasting and more intense blocking [4].

The alpha-2 agonist dexmedetomidine, which is composed of the dextrorotatory enantiomer of medetomidine, is regarded as a prototype for highly selective alpha-2 adrenergic agonists [5]. As an adjuvant, it intensifies motor block and increases the duration of postoperative pain relief [6]. Dexmedetomidine produces sedation without significant respiratory depression [7]. Clonidine is also an alpha-2 adrenergic agonist, which has analgesic properties and potentiates local anaesthetic effects [8]. It is known to have sedative properties, and the side effects are hypotension and bradycardia. Nalbuphine, a derivative of 14-hydroxy morphine, is an analgesic with partial agonist-antagonist activity with strong k-receptor agonism and weak μ-receptor agonist and antagonist activity [9]. They have strong analgesic potency and sedative properties with a ceiling effect on mu (μ)-receptor activity of respiratory depression.

In this study, our primary objectives were to compare the onset and duration of sensory and motor blockade and the duration of analgesia, while our secondary objectives were to compare the onset and duration of sedation, haemodynamic changes, and side effects and complications in nalbuphine, clonidine, and dexmedetomidine as an adjuvant to 0.75% ropivacaine in epidural anaesthesia in lower limb surgery.

## Materials and methods

With the approval of the ethical committee of Uttar Pradesh University of Medical Sciences (UPUMS), Saifai, Etawah, India (Ref no: 496/UPUMS/DSW/Ethical/2022-2023, Clearance no: 47/2022-23, CTRI number: CTRI/2024/02/062736), we conducted a prospective randomised double-blind study from 1 March 2024 to 30 September 2024. We included 90 patients aged 18-65 years of the American Society of Anaesthesiologists' (ASA) grade I and II, with a BMI of 18-30 kg/m^2^ of either sex posted for lower limb orthopaedic surgery. Patients with coagulopathy/thrombocytopenia, localised infection, opioid tolerance/dependence, renal/hepatic impairment, known cardiorespiratory impairment, pregnant women, women and those allergic to drugs used in the study, and breastfeeding women were excluded.

Sample size: The sample size has been calculated by using the following formula.

n= [{z_(1-α/2)_+z_(1-β)_}^2^x(2σ^2^)]/(µ_1_-µ_2_)^2^

n= sample size required in each group; z_(1-α/2)_ = standardised normal deviate (two-tailed); at α=0.05 & 95%CL it is 1.96; z_(1-β)_ = at 80% power of the study, it is 0.84; while σ2=[(N_1_-1)S_1_^2^+(N_2_-1)S_2_^2^]/[(N_1_-N_2_)-2]; N1&S1 = sample size and standard deviation (SD)of one group and N2 & S2 = sample size and standard deviation

Methodology

After taking informed written consent, all 90 patients were randomly divided into three groups: Group D (received 15 ml of 0.75% ropivacaine + injection (inj.) dexmedetomidine 1.5 μg/kg + normal saline to make it a total volume of 18 ml), Group N (received 15 ml of 0.75% ropivacaine + inj. nalbuphine 0.2 mg/kg + normal saline to make it a total volume of 18 ml), and Group C (received 15 ml of 0.75% ropivacaine + inj. clonidine 1.5 μg/kg + normal saline to make it a volume of 18 ml). A random number table for 90 patients, divided into three groups (30 patients each), was generated, and sequentially numbered opaque sealed envelopes were prepared. The anaesthesiologist who administered the block was not involved in encoding the data, and the observer who recorded all pain scores was also blinded to the used method. After complete preoperative evaluation. Intravenous access was achieved with an 18G cannula, and each patient was pre-loaded with 10 ml/kg body weight Ringer’s lactate solution 10 minutes before induction of epidural analgesia. With proper aseptic and antiseptic precautions, under local anaesthesia (2% lignocaine), a multi-hole epidural catheter (Prisafe Safety First Epidural Catheter Kit, Iscon Surgicals Limited, Jodhpur, India) was inserted at the L3-L4 intervertebral space with an 18G Touhy needle using the loss of resistance technique to air. The catheter was placed 3-4 cm in the epidural space in the cephalic direction. A test dose of 3 ml of 2% lignocaine with 1:200,000 epinephrine was administered to exclude intravenous or subarachnoid catheter placement after negative aspiration for cerebrospinal fluid (CSF) or blood along with the absence of tachycardia. Epidural anaesthesia was given in different groups using respective drugs as discussed before.

Monitoring was done up to the first 24 hours from the time of anaesthesia and was noted. The following parameters, viz., heart rate (HR), systolic blood pressure (SBP), diastolic blood pressure (DBP), mean arterial blood pressure, oxygen saturation, and respiratory rate, were also recorded at the baseline, before the block, at the time of the block (0 minute), the fifth minute, the 10^th^ minute, the 15^th^ minute, the 20^th^ minute, the 25^th^ minute, the 30^th^ minute, the 45^th^ minute, the first hour, one hour 15 minutes, one hour 30 minutes, one hour 45 minutes, two hours, two hours 15 minutes, two hours 30 minutes, two hours 45 minutes, three hours, six hours, 12 hours, and 24 hours during the intraoperative and postoperative period. Onset time to T 10 level was taken as the time interval between the end of total local anaesthetic administration (time zero) and complete sensory block at T 10 level (pinprick test score 2). The duration of sensory block is defined as the time interval between the end of total local anaesthetic administration (time zero) and the complete resolution of anaesthesia (pinprick test score 0). Motor block onset time is the time interval between the zero time and the Modified Bromage scale (MBS score ≥2). The duration of the motor block is the time interval from the onset to the recovery of complete motor function (MBS score 0). Sedation was assessed by Ramsay's Sedation Score (RSS). Pain was assessed by the visual analogue scale (VAS). Patients with VAS >3 received an intramuscular injection of diclofenac (1.5 mg/kg), and if this was not sufficient, then tramadol (1 mg/kg) was given intravenously. Diclofenac up to a maximum of 150 mg and tramadol up to a maximum of 400 mg were administered in 24 hours. Epidural analgesia top-ups were not given to patients to eliminate errors in measuring other outcomes of the studies. Adverse events such as bradycardia (heart rate (HR) <60 beats per minute (bpm) or a 20% drop from the baseline value), tachycardia (HR increase more than 20% from baseline), hypotension (a 20% drop in blood pressure from the baseline or an absolute mean arterial pressure (MAP) <60 mmHg), and hypertension (MAP > 100 mmHg). Clinically significant bradycardia (HR < 50 bpm) was treated with increment doses of injected atropine 0.6 mg intravenously. A bolus of IV crystalloids or incremental injections of 6 mg of mephentermine intravenously were used to treat hypotension. Data was analysed using IBM SPSS Statistics software, version 23.0 (IBM Corp., Armonk, NY, USA). Mean, standard deviation, ANOVA, chi-square test, and unpaired T-test were applied, and p-value < 0.05 was found to be statistically significant.

## Results

**Figure 1:**
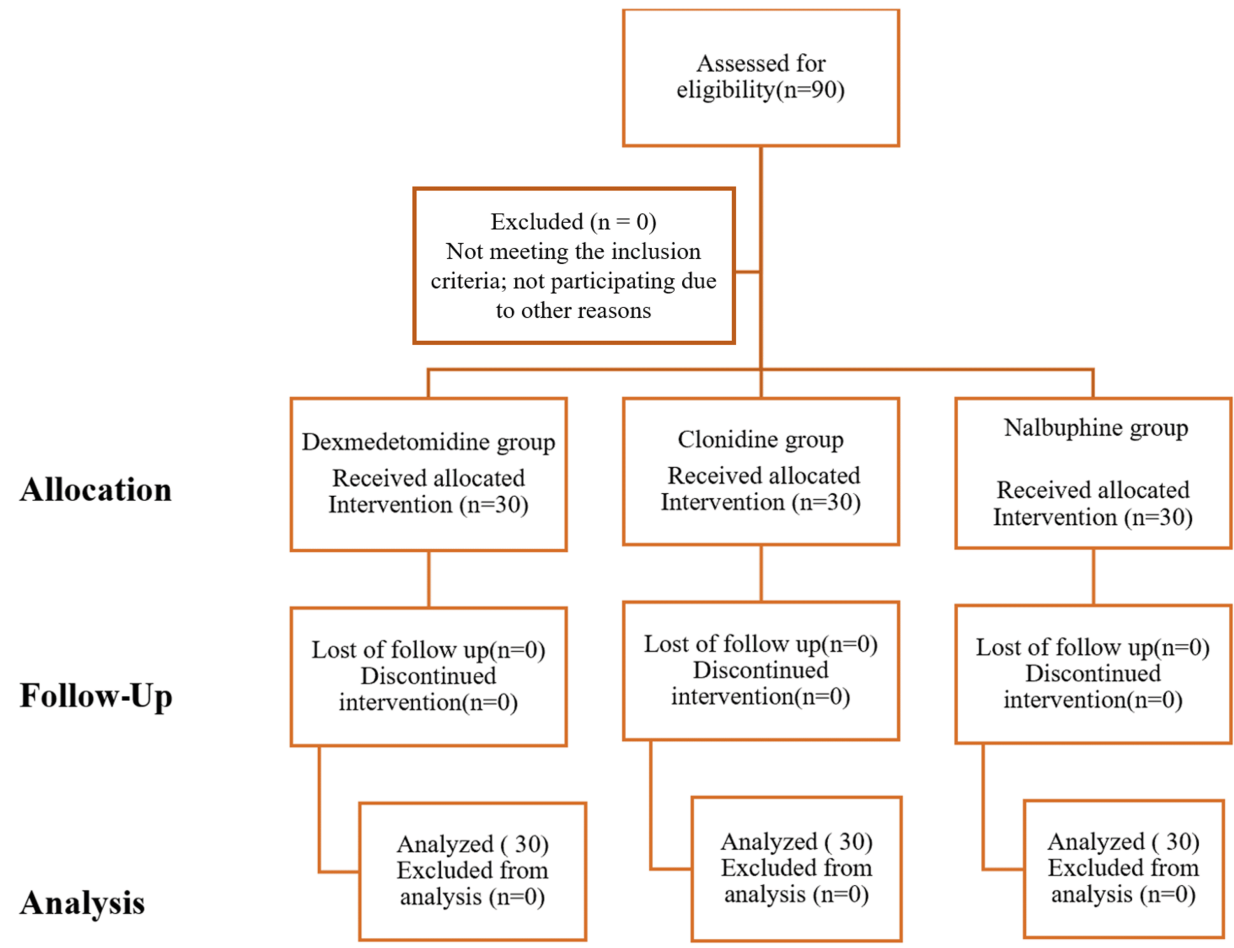
Consolidated Standards of Reporting Trials (CONSORT) diagram n= number of patients

We included 90 patients (30 patients in each group); none were excluded, as shown in the Consolidated Standards of Reporting Trials (CONSORT) diagram (Figure 1).

The demographic profile was comparable in all the groups in regard to age, weight, height, BMI, and duration of surgery (p-value >0.05; Table 1).

**Table 1:** Participants' demographic details (age, weight, height, BMI, and duration of surgery) ANOVA test used for statistical analysis; n = number of patients, t-value, F-value; *p-value < 0.05, statistically significant, SD = standard deviation; Group D = dexmedetomidine group; Group N= nalbuphine group; Group C = clonidine group

Groups	Group D (n=30)	Group C (n=30)	Group N (n=30)	p-value		
	Mean ±SD	Mean ±SD	Mean ±SD	D vs C	D vs N	C vs N
Age (years)	34.1±12.6	39.0±14.4	37.9±13.4	0.348	0.529	0.947
Weight (kg)	67.7±8.6	68.6±8.9	68.3±8.3	0.159	0.159	0.992
Height (cms)	169.7±8.1	168.5±7.5	168.7±8.7	0.819	0.883	0.991
BMI (kg/m^2^)	25.2±2.4	24.1±2	23.9±1.5	0.474	0.136	0.717
Duration of surgery (min)	92.3±22.8	96.9±22.3	103.1±24.5	0.430	0.082	0.312

The time taken to achieve the sensory block at the T10 level was the least in Group D (8.0±1.1 minutes) and Group C (10.3±1.4 minutes) and highest in Group N (11.3±1.5 minutes). In comparison between the groups, the mean values were found to be very highly statistically significant (p < 0.001) (Table 2). The total duration of sensory block was highest in Group D (495.5±16.1 minutes) and Group N (356.8±17.7 minutes) and shortest in Group C (309.9±13.4 minutes), found to be very highly statistically significant. (p < 0.001; Table 2).

**Table 2:** Onset and duration of sensory, motor blockade and sedation, and duration of analgesia ANOVA test used for statistical analysis; n = number of patients, t-value, F-value; *p-value < 0.05, statistically significant; SD = standard deviation; Group D = dexmedetomidine group; Group N = nalbuphine group; Group C = clonidine group

		Group D (n=30) (Mean±SD)	Group C (n=30) (Mean±SD)	Group N (n=30) (Mean±SD)	Group D vs. Group C		Group D vs. Group N		Group C vs. Group N		Overall	
					t-value	p-value	t-value	p-value	t-value	p-value	F-value	p-value
Sensory blockade (minutes)	Onset	8.0±1.1	10.3±1.4	11.3±1.5	-7.053	<0.001	-10.164	<0.001	-2.790	<0.001	50.173	< 0.001*
	Duration	495.5±16.1	309.9±13.4	356.8±17.7	48.546	<0.001	31.779	<0.001	-11.594	<0.001	1117.252	< 0.001*
Motor blockade (minutes)	Onset	10.5±1.7	14.7±1.1	14.8±1.4	-11.410	<0.001	-10.870	<0.001	-0.302	0.763	90.438	< 0.001*
	Duration	405.7±16	255.7±11	257±13.4	42.378	<0.001	39.132	<0.001	-0.412	0.682	1207.440	< 0.001*
Sedation (minutes)	Onset	32.7±5.1	39.7±8.5	34.4±5.6	-2.190	0.044*	-0.292	0.046*	1.674	0.120	2.778	0.083
	Duration	53.5±8.6	31.3±5.8	34.3±6.3	5.658	<0.001*	5.413	< 0.001*	-0.891	0.390	25.093	< 0.001*
Analgesia (minutes)	Duration	525.5±16.1	340.0±13.4	386.8±17.6	48.546	<0.001*	31.779	< 0.001*	-11.594	<0.001*	1117.252	< 0.001*

Time taken to reach the complete motor block was minimum in Group D (10.5±1.7 minutes) and Group C (14.7±1.1 minutes) and highest in Group N (14.8±1.4 minutes), found to be very highly statistically significant (p<0.001) in Group D as compared to Group C and in Group D as compared to Group N. While mean values of onset of motor block are comparable in Group C and Group N, which were statistically not significant (p > 0.05) (Table 2). The total duration of motor block was longest in Group D (405.7±16 minutes) and Group N (257±13.4 minutes) and shortest in Group C (255.7±11 minutes), found to be statistically very highly significant (p<0.001) in Group D as compared to Group C and Group D as compared to Group N. while the mean values of duration of motor block were comparable in Group C and Group N, which were statistically not significant (p > 0.05; Table 2).

The duration of analgesia was maximum in Group D (525.5±16.1 minutes), Group N (386.8±17.6 minutes), and least in Group C (340.0±13.4 minutes). Intercomparison among the group was found to be statistically very highly significant. (p < 0.001) (Table 2).

**Figure 2:**
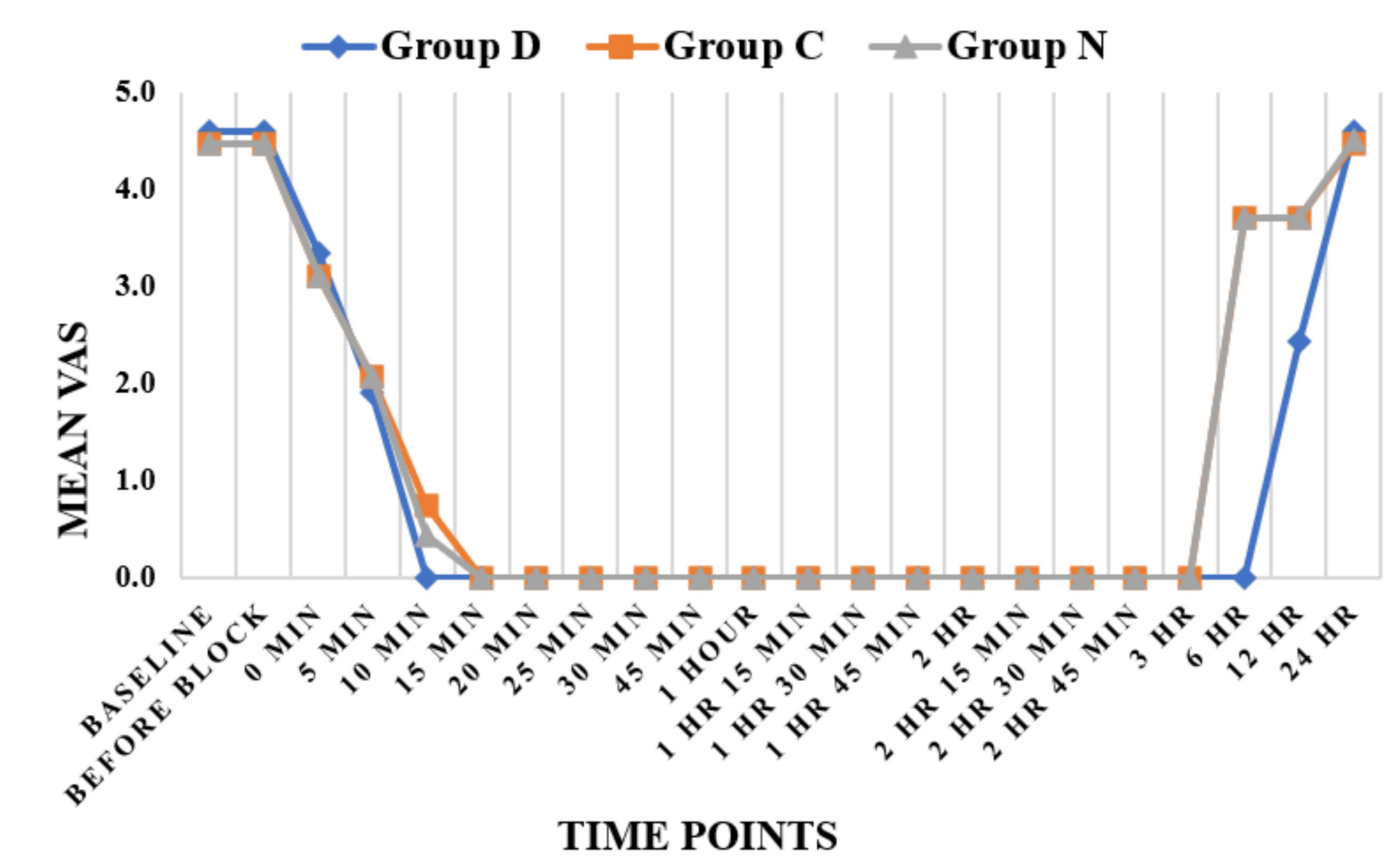
Inter-group comparison for changes in the visual analogue scale at different time intervals perioperatively from baseline up to 24 hours of surgery Group D = dexmedetomidine group; N = nalbuphine group; Group C = clonidine group

Figure 2 shows an inter-group comparison for changes in the visual analogue scale at different time intervals perioperatively from baseline up to 24 hours of surgery. Baseline VAS in Groups D, C, and N were 4.6±0.77, 4.47±0.51, and 4.47±0.51, respectively, and were found to be comparable (p > 0.05). After 10 minutes, patients experienced more pain in Group C (VAS 0.73±0.87) and Group N (VAS 0.43±0.5) as compared to no pain in Group D (VAS 0), which was due to complete analgesia in patients receiving dexmedetomidine at 10 min, which was statistically very highly significant (p-value <0.001). At six hours, patients experienced pain in Group C (VAS 3.7±0.65) and Group N (VAS 3.7±0.65), while there was no pain in Group D (VAS 0), which was statistically very highly significant (p-value <0.001). After six hours, up to 12 hours, patients experienced pain gradually once the analgesic effect of groups disappeared. Overall, the VAS score was lower in the dexmedetomidine group.

Out of 30 patients in each group, 12 patients had sedation (RSS ≥3) in Group D, six patients had sedation (RSS ≥3) in Group C, and eight patients had sedation (RSS ≥3) in Group N. Mean onset of sedation was early in Group D (32.7±5.1 minutes) as compared to Group C (39.7±8.5 minutes) and Group N (34.4±5.6 minutes), found to be statistically significant (p=0.04). The duration of sedation was maximum in Group D (53.5±8.6 minutes) and Group N (34.3±6.3 minutes) and least in Group C (31.3±5.8 minutes). Intercomparison among the group was found to be statistically very highly significant. (p < 0.001) in Group D as compared with Group N and Group C, while onset and duration of sedation were comparable in Group C and Group N (p-value > 0.05; Table 2).

Total diclofenac requirement was minimum in Group D (102.50±36.76 mg) and Group N (147.50±13.69 mg) and maximum in Group C (150.00±00 mg), found to be statistically highly significant (p<0.001). Total tramadol requirement was minimum in Group D (100.00±0 mg) and Group N (168.00±80.21 mg) and maximum in Group C (270.00±74.97 mg), found to be statistically significant (p < 0.036) in Group D as compared to Group N, while statistically very highly significant in Group C as compared to Group D and Group N (p < 0.001). The demand for diclofenac as rescue analgesia was the same in Groups N and C, but the demand for tramadol was greater in Group C and Group N as compared to Group D. The total dose of the analgesic requirement was greater in Group C as compared to the remaining two groups (p < 0.001). The analgesic requirement was lowest in Group D (Table 3).

**Table 3:** Total dose of analgesic requirement ANOVA test used for statistical analysis; n = number of patients, t-value, F-value; *p-value < 0.05, statistically significant; SD = standard deviation; Group D = dexmedetomidine group; Group N = nalbuphine group; Group C = clonidine group

Total analgesics in 24 hours	Group D (n=30) (Mean±SD)	Group C (n=30) (Mean±SD)	Group N (n=30) (Mean±SD)	Group D vs. Group C		Group D vs. Group N		Group C vs. Group N		Overall	
				t-value	p-value	t-value	p-value	t-value	p-value	F-value	p-value
Diclofenac (mg)	102.5±36.7	150.0±00	147.5±13.69	7.077	<0.001	6.283	<0.001	-1.000	<0.001	47.238	<0.001
Tramadol (mg)	100.0±0	270.0±74.97	168.0±80.21	12.415	0.036	4.643	<0.001	-5.088	<0.001	35.291	<0.001

Bradycardia was observed in four patients in Group D, 10 in Group C, and two in Group N, and found to be statistically significant among the groups (p = 0.019). Hypotension, nausea, and vomiting were also observed in patients in Group D, Group C, and Group N, found not to be statistically insignificant (p = 0.338). None of the patients develop tachycardia, hypertension, or itching in any group (Table 4).

**Table 4:** Adverse effects Chi-square test used for statistical analysis; n = number of patients; *p-value < 0.05, statistically significant; SD = standard deviation; Group D = dexmedetomidine group; Group N = nalbuphine group; Group C = clonidine group

Adverse events	Group D (n=30)		Group C (n=30)		Group N (n=30)		Total (n=90)		p-value
	n	%	n	%	N	%	n	%	
Nausea	5	16.7%	8	26.7%	7	23.3%	20	22.2%	0.638
Vomiting	3	10.0%	4	13.3%	3	10.0%	10	11.1%	0.894
Bradycardia	4	13.3%	10	33.3%	2	6.7%	16	17.8%	0.019*
Tachycardia	0	0.0%	0	0.0%	0	0.0%	0	0.0%	-
Hypotension	1	3.3%	4	13.3%	2	6.7%	7	7.8%	0.338
Hypertension	0	0.0%	0	0.0%	0	0.0%	0	0.0%	-
Itching	0	0.0%	0	0.0%	0	0.0%	0	0.0%	-

Hemodynamic parameters

**Figure 3:**
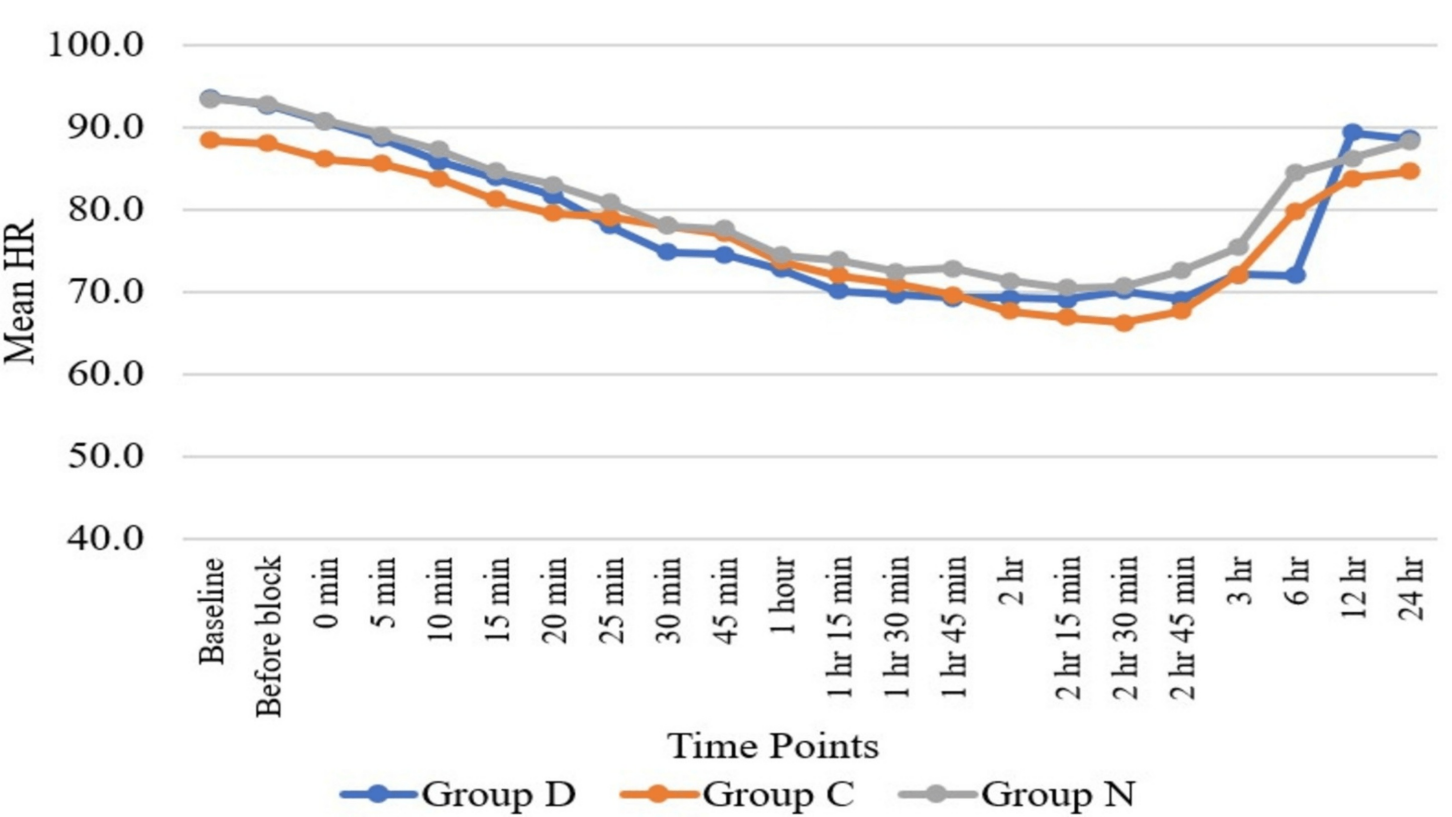
Comparison of heart rate (HR) with time Heart rate= beats per minute; Group D = dexmedetomidine group; Group N = nalbuphine group; Group C = clonidine group

**Figure 4:**
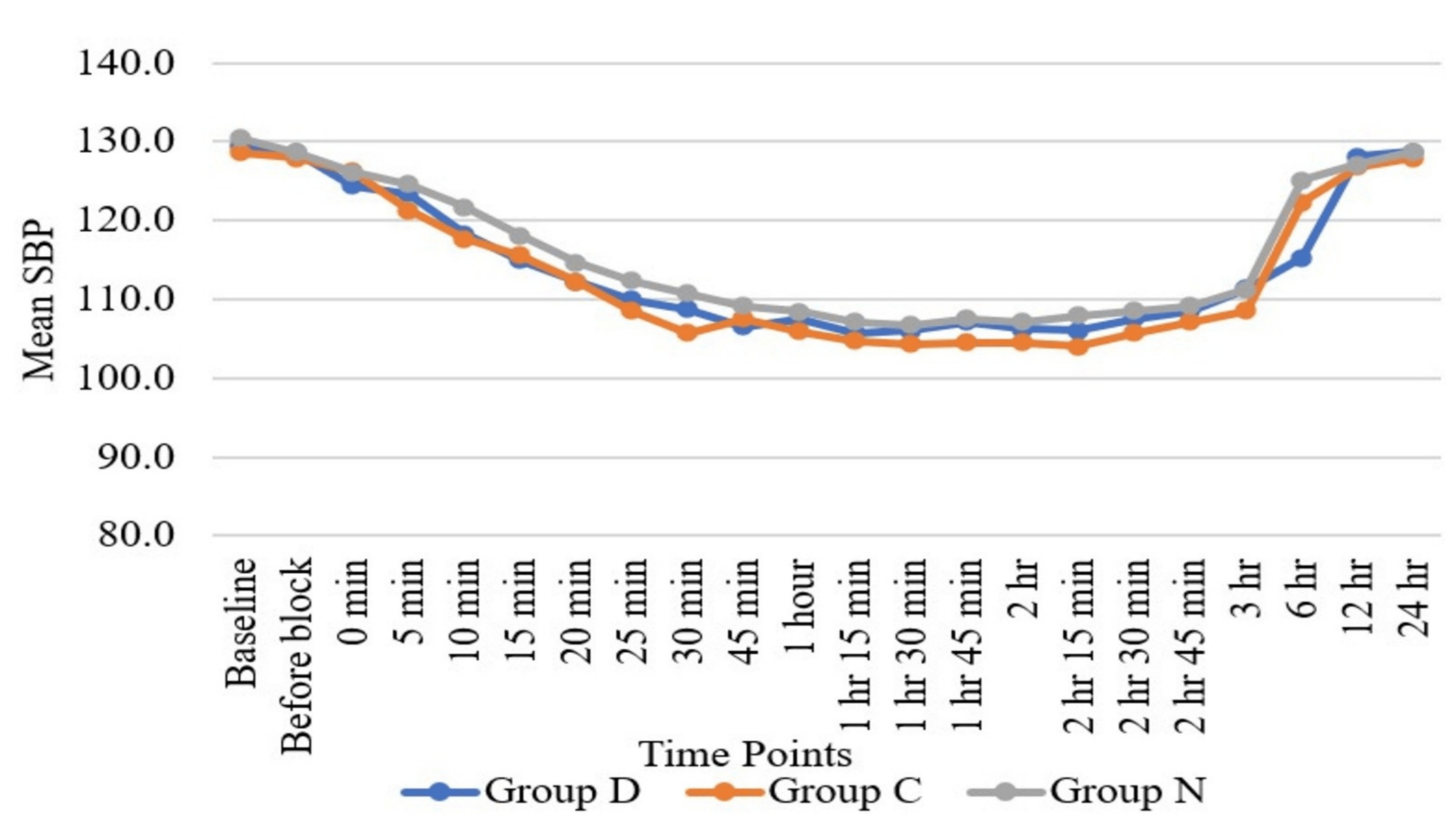
Comparison of systolic blood pressure (SBP) with time SBP = mmHg; Group D = dexmedetomidine group; Group N = nalbuphine group; Group C = clonidine group

**Figure 5:**
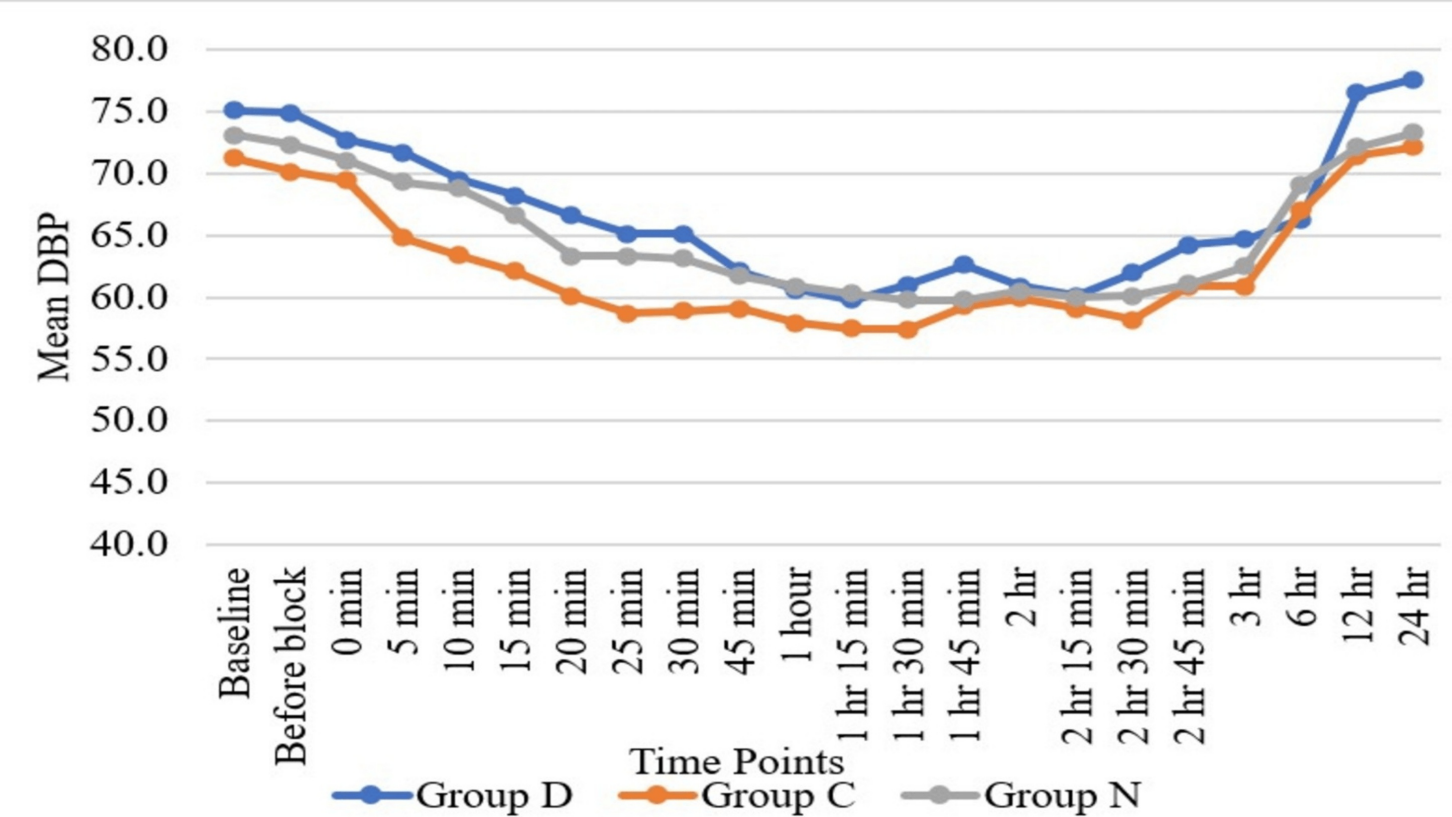
Comparison of diastolic blood pressure (DBP) with time DBP = mmHg; Group D = dexmedetomidine group; Group N = nalbuphine group; Group C = clonidine group

**Figure 6:**
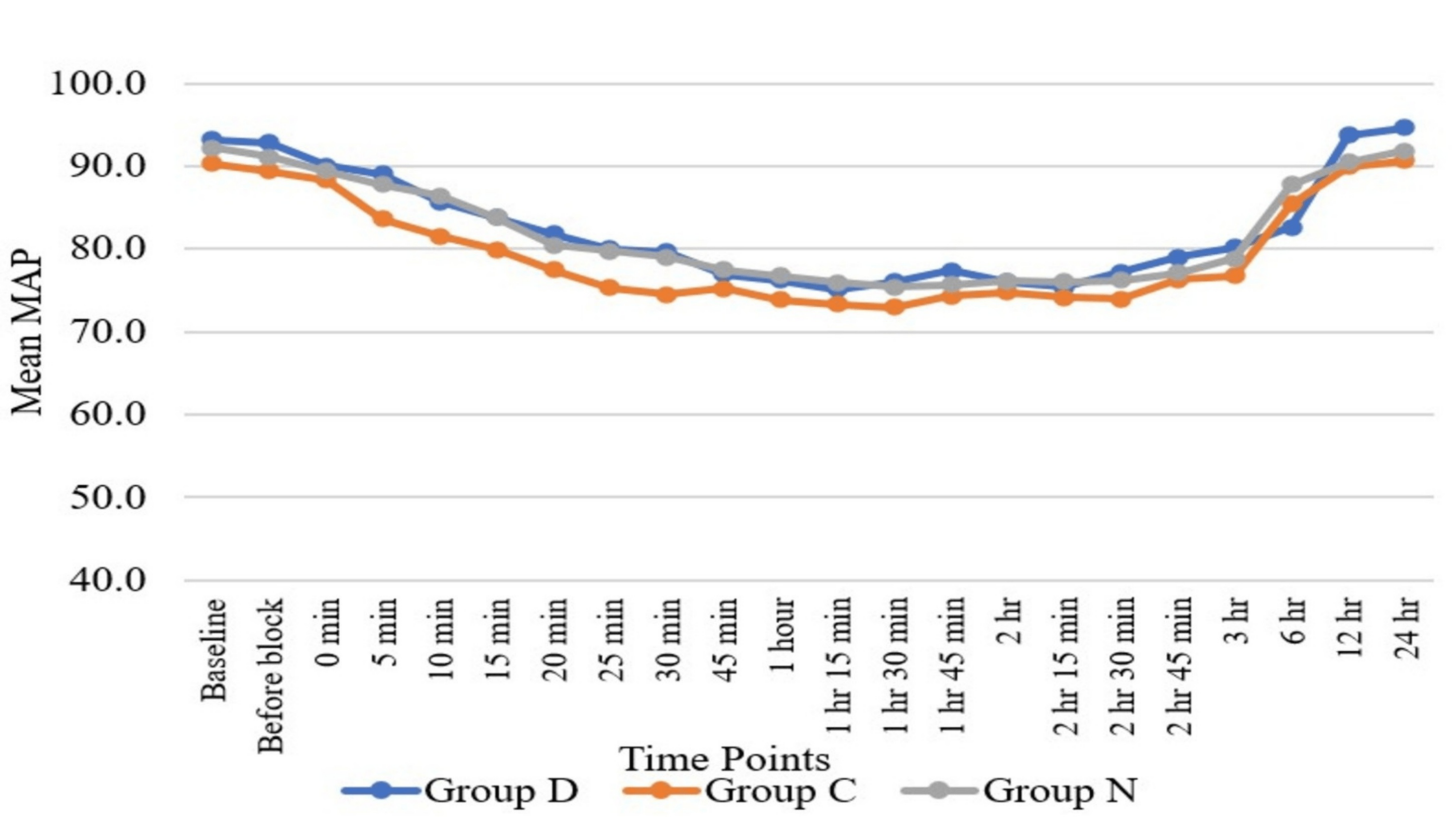
Comparison of mean arterial pressure (MAP) with time MAP = mmHg; Group D = dexmedetomidine group; Group N = nalbuphine group; Group C = clonidine group

**Figure 7:**
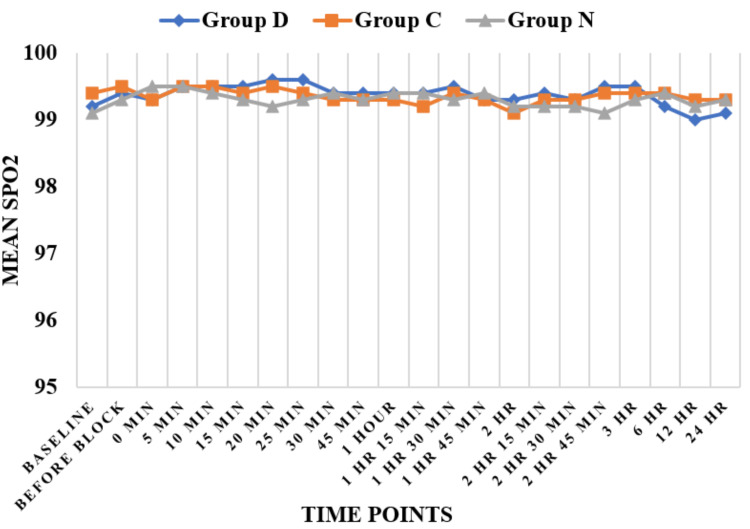
Oxygen saturation (SpO2) SpO_2_ : % of oxygen saturation; Group D = dexmedetomidine group; Group N = nalbuphine group; Group C = clonidine group

**Figure 8:**
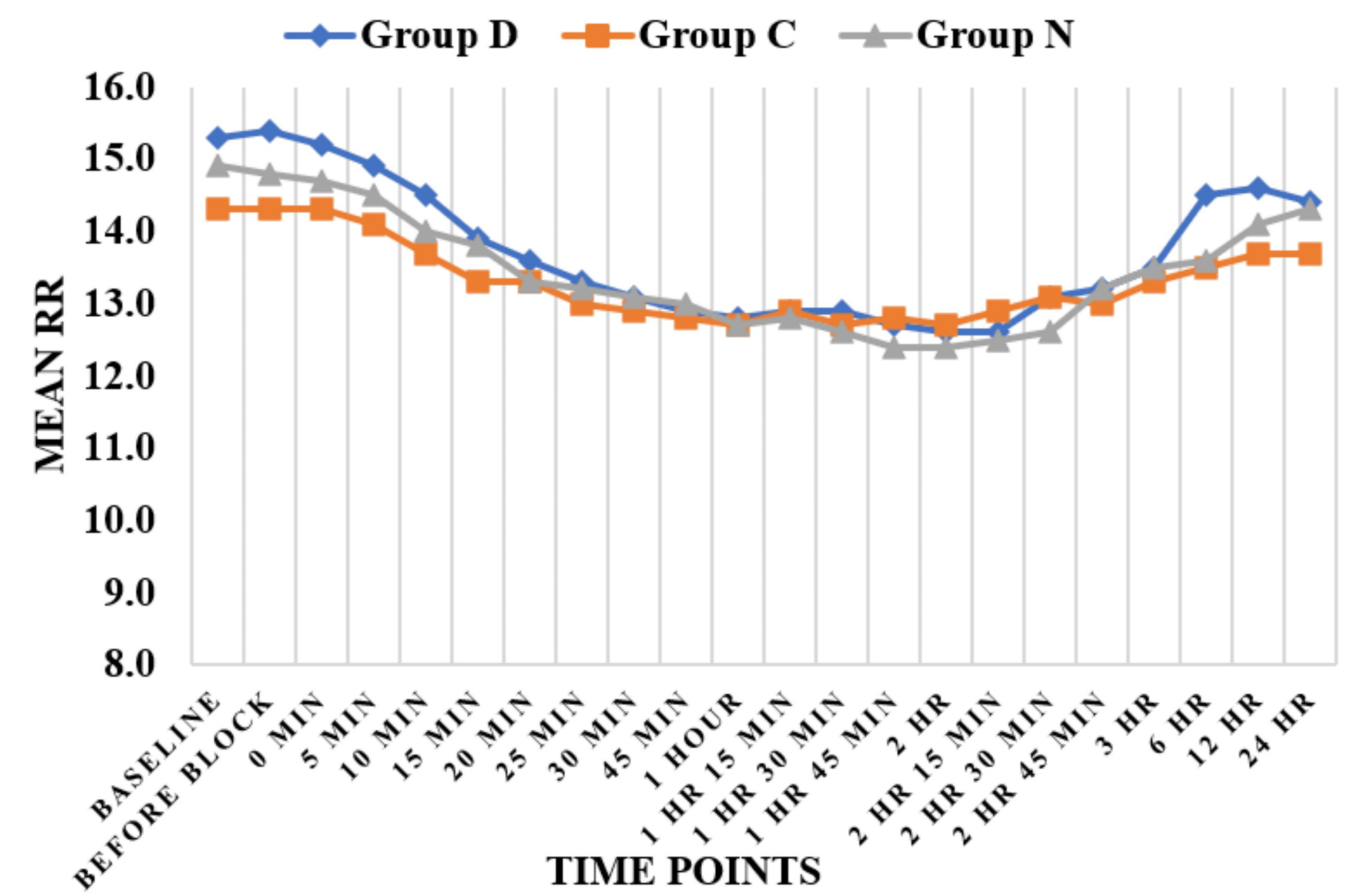
Respiratory rate (RR) Group D = dexmedetomidine group; Group N = nalbuphine group; Group C = clonidine group

Haemodynamic parameters like HR (Figure 3) and SBP (Figure 4) showed statistically significant differences in Group D as compared to Group N and Group C at six hours, while HR and SBP were comparable among all three groups at all other time periods except at six hours. Regarding DBP (Figure 5), DBP was comparable among all three groups at all the time periods except five minutes, 10 minutes, 15 minutes, 20 minutes, 25 minutes, and 30 minutes. At five minutes, 10 minutes, 15 minutes, 20 minutes, 25 minutes, and 30 minutes, the clonidine group showed a significant fall in diastolic blood pressure as compared to the dexmedetomidine group. Mean arterial pressure was comparable among all three groups at all the time periods except 20 minutes, 25 minutes, and 30 minutes (Figure 6). At 20 minutes, 25 minutes, and 30 minutes, the clonidine group showed a significant fall in mean arterial blood pressure as compared to the dexmedetomidine group. This is due to a significant fall in DBP at 20 minutes, 25 minutes, and 30 minutes as compared to the dexmedetomidine group, leading to a significant fall in MAP at these time periods. while haemodynamic parameters like oxygen saturation (SpO_2_) (Figure 7) and RR (Figure 8) were comparable among all three groups at all time periods.

## Discussion

Chandran et al. [4] compared the characteristics of 0.75% ropivacaine and 0.5% bupivacaine and concluded that ropivacaine and bupivacaine at these doses produced equally effective anaesthesia; 0.75% ropivacaine produces adequate intensity of motor and sensory blockade and is comparable to 0.5% bupivacaine with reduced side effects. Hence, we used 0.75% ropivacaine to provide epidural anaesthesia.

The onset of sensory was shortest in Group D (8.0±1.1 minutes) and Group C (10.3±1.4 minutes) and longest in Group N (11.3±1.5 minutes), found to be very highly statistically significant. (p<0.001) while the total duration of sensory block was highest in Group D (495.5±16.1 minutes) and Group N (356.8±17.7 minutes) and shortest in Group C (309.9±13.4 minutes), found to be very highly statistically significant. (p < 0.001. Farmawy et al. [10] (2023) also found the onset of sensory block was early and duration was prolonged in the dexmedetomidine group (12.17 ± 1.27 minutes and 365.87 ± 18.01 minutes) as compared to the nalbuphine group (13.39 ± 1.27 minutes and 318.38 ± 22.54 minutes), supporting our study. Onset was delayed in dexmedetomidine as well as nalbuphine, as they used a lower concentration of 0.25% bupivacaine as compared to 0.75% ropivacaine used in our study. Arunkumar et al. [11] (2015) also found that the onset of sensory block was early and duration was prolonged in the dexmedetomidine group (8.53 ± 1.81 min and 316 ± 31.15 min) as compared to the clonidine group (11.93 ± 1.96 min and 281 ± 37 minutes). The duration was shorter in both groups than in our study because they used lower concentrations of dexmedetomidine (1 mcg/kg) and clonidine (1 mcg/kg) than we used.

Time taken to reach the complete motor block was minimum in Group D (10.5±1.7 minutes) and Group C (14.7±1.1 minutes) and highest in Group N (14.8±1.4 minutes), found to be very highly statistically significant (p < 0.001), while total duration of motor block was longest in Group D (405.7±16 minutes), Group N (257±13.4 minutes), and shortest in Group C (255.7±11 minutes), found to be statistically very highly significant (p < 0.001) in Group D as compared to Group C and Group D as compared to Group N. Mean values of duration of motor block were comparable in Group C and Group N, which were statistically not significant (p > 0.05). Arunkumar et al. [11] (2015) also found delayed and no statistically significant difference in time to complete and duration of motor blockade between the dexmedetomidine group (23 minutes and 161±00 minutes) and clonidine group (23 minutes and 138.17±00 minutes); this may be due to their use of lower concentrations of 1 mcg/kg dexmedetomidine and 1 mcg/kg clonidine as compared to our study, where we found a statistically significant difference at a higher dose of 1.5 mcg/kg dexmedetomidine and 1.5 mcg/kg of clonidine given with 0.75% ropivacaine.

Baseline VAS in Groups D, C, and N were 4.6±0.77, 4.47±0.51, and 4.47±0.51, respectively, and were found to be comparable (p > 0.05). After 10 minutes, patients experienced more pain in Group C (VAS 0.73±0.87) and Group N (VAS 0.43±0.5) as compared to no pain in Group D (VAS 0), which was due to complete analgesia in patients receiving dexmedetomidine at 10 min, which was statistically very highly significant (p-value <0.001). At 360 minutes, patients experienced pain in Group C (VAS 3.7±0.65) and Group N (VAS 3.7±0.65), while there was no pain in Group D (VAS 0), which was statistically very highly significant (p-value <0.001). After 360 minutes up to 720 minutes, patients experienced pain gradually once the analgesic effect of Group D disappeared. Overall, the VAS score was lower in Group D as compared to Group N and Group C. Shaikh et al. [12] (2017) also found that the dexmedetomidine group's VAS score began to rise only after 390 minutes and reached its maximum at 450-480 minutes; the clonidine group's VAS score was greater, necessitating rescue analgesia at 300 minutes and reaching its highest at 360-390 minutes. The VAS scores were lower in the dexmedetomidine group even at 360, 390, and 420 minutes, consistent with our study, as we found a significant rise in VAS scores after six hours (360 minutes). Bajwa et al. [8] (2011) also found that the VAS score begins to rise once the sensory effect starts weaning off, while the time for first rescue analgesia (VAS > 3) was longer in dexmedetomidine as compared to clonidine. Overall, the VAS score was lower in Group D than in Group C, which is similar to our study. Arunkumar et al. [11] also found that the VAS score was lower in the dexmedetomidine group as compared to the clonidine group; the results were in favour of our study.

The total duration of analgesia was longest in Group D (525.5±16.1 minutes) and Group N (386.8±17.6 minutes) and shortest in Group C (340.0±13.4 minutes), found to be very highly statistically significant. (p < 0.001). Arunkumar et al. [11] found favourable results in view of the duration of analgesia; they found the duration was prolonged in patients receiving dexmedetomidine (316 ± 31.15 min) as compared to clonidine (281 ± 37 min), while the duration of analgesia was less in both groups as compared to our groups. It may be due to their use of lower concentrations of dexmedetomidine (1 mcg/kg) and clonidine (1 mcg/kg).

The total dose of the analgesic requirement was greater in Group C as compared to the remaining two groups (p < 0.001). The analgesic requirement was lowest in Group D. Farmawy et al. [10] (2023) also found that total top-up doses and postoperative analgesia (they used ketorolac maximum up to 60 mg) requirements were more in Group N as compared to Group D, supporting our study. Kabi et al. [13] (2021), and Shaikh et al. [12] (2017), also found that the clonidine group required more postoperative doses of analgesics as compared to the dexmedetomidine group, favouring our study.

Out of 30 patients, 12 patients in Group D, six in Group C, and eight patients in Group N had sedation (RSS > 3). The clonidine group showed significant bradycardia, hypotension, nausea, and vomiting as compared to the others. None of the patients develop tachycardia, hypertension, and itching. Farmawy et al. [10] found that eight patients in the dexmedetomidine group, which was more as compared to one patient in the nalbuphine group, developed bradycardia, which was statistically significant as found to be similar to our study. As far as nausea, vomiting, and hypotension results were concerned, they were comparable in the nalbuphine and dexmedetomidine groups, similar to our study. Kabi et al. [13], the side effects of clonidine included nausea, which was observed in two cases but was not statistically significant, whereas hypotension, bradycardia, motor involvement, and nausea were observed in one case each in the dexmedetomidine group. Arunkumar et al. [11] found no significant differences in terms of hypotension, nausea, and vomiting between the patients receiving dexmedetomidine or clonidine, similar to our study.

Haemodynamic parameters like HR, SBP, SpO_^2^_, and RR were comparable among all three groups at all time periods except at six hours, where HR (Figure 2) and SBP (Figure 3) showed significant statistical differences between Group D and Group N. Regarding DBP (Figure 4), DBP was comparable among all three groups at all time periods except five minutes, 10 minutes, 15 minutes, 20 minutes, 25 minutes, and 30 minutes, where the clonidine group showed a significant fall in diastolic blood pressure as compared to the dexmedetomidine group. Regarding MAP (Figure 5), MAP was comparable among all three groups at all time periods except 20 minutes, 25 minutes, and 30 minutes, where the clonidine group showed a significant fall in mean arterial blood pressure due to a significant fall in DBP in the same time period as compared to the dexmedetomidine group. Unlike other studies, such as those by Farmawy et al. [10], Kabi et al. [13], and Arunkumar et al. [11], they found insignificant results in haemodynamic parameters. Ultrasound (USG)-guided regional nerve blocks are a newer, safer, and more effective postoperative pain control modality for elderly patients [14]. The study findings can also be utilised in the formulation of a uniform and structured pain management policy at the institutional level [15].

Limitation

We did not measure the levels of dexmedetomidine, clonidine, and nalbuphine in the plasma which could have further supported the hypothesis, and the subjectivity of VAS for pain assessment with a variable level of understanding between patients was a limitation of our study.

## Conclusions

From this study, it can be concluded that the use of dexmedetomidine as an adjuvant to the local anaesthetic agent during epidural block hastens the onset of sensory and motor blockade, provides a longer duration of sensory and motor block, provides a longer duration of analgesia, and decreases the total analgesic requirement without causing clinically significant and unmanageable side effects as compared to nalbuphine followed by clonidine.
